# Supplementary material for: Application of Exogenous Ascorbic Acid Enhances Cold Tolerance in Tomato Seedlings through Molecular and Physiological Responses
Source: Int J Mol Sci. 2024 Sep 19;25(18):10093. doi: 10.3390/ijms251810093 (PMC11432314; doi:10.3390/ijms251810093)
Supplement: Supplementary file 1 [file ijms-25-10093-s001.zip › Supplementary Figures.pdf]

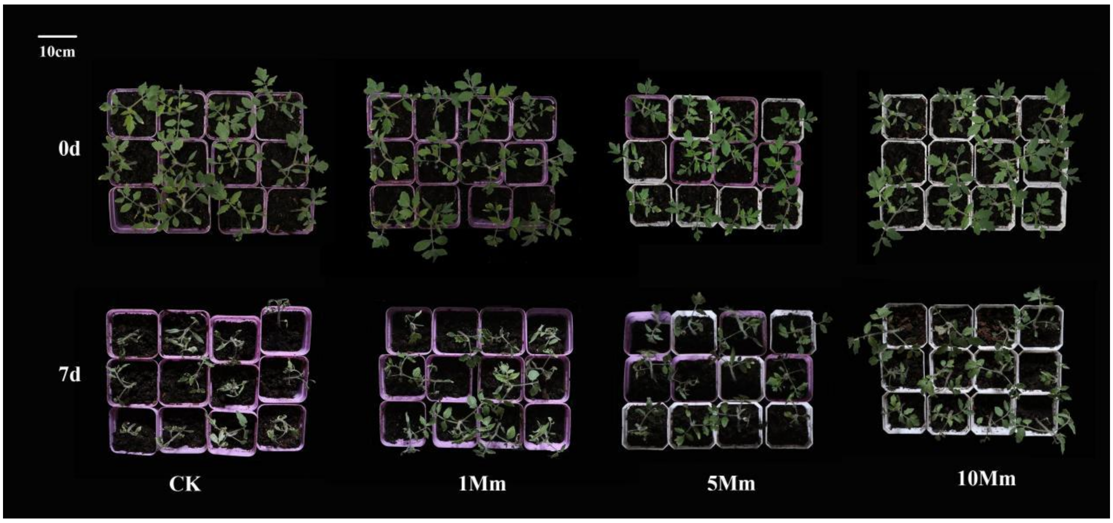

Figure S1. Phenotype of different solutions AsA-pretreated tomato seedlings.

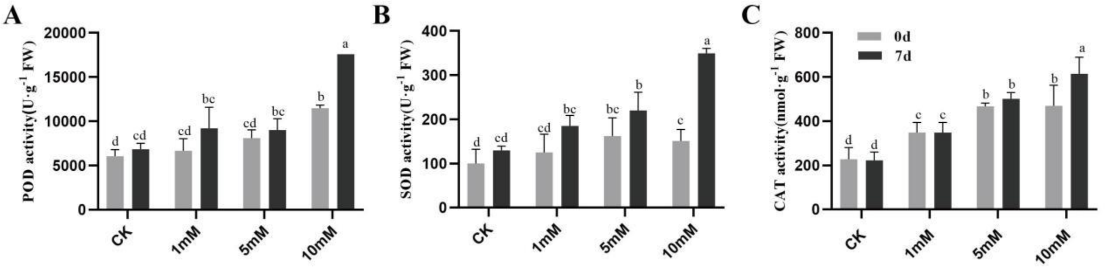

Figure S2. Effect of exogenous AsA application on antioxidant enzyme activity in tomato. (**A**) POD activity. (**B**) SOD activity. (**C**) CAT activity.
